# *In Silico* Prediction of Human Pathogenicity in the γ-Proteobacteria

**DOI:** 10.1371/journal.pone.0013680

**Published:** 2010-10-27

**Authors:** Massimo Andreatta, Morten Nielsen, Frank Møller Aarestrup, Ole Lund

**Affiliations:** 1 Center for Biological Sequence Analysis, Technical University of Denmark, Kongens Lyngby, Denmark; 2 National Food Institute, Technical University of Denmark, Copenhagen, Denmark; Institut de Pharmacologie et de Biologie Structurale, France

## Abstract

**Background:**

Although the majority of bacteria are innocuous or even beneficial for their host, others are highly infectious pathogens that can cause widespread and deadly diseases. When investigating the relationships between bacteria and other living organisms, it is therefore essential to be able to separate pathogenic organisms from non-pathogenic ones. Using traditional experimental methods for this purpose can be very costly and time-consuming, and also uncertain since animal models are not always good predictors for pathogenicity in humans. Bioinformatics-based methods are therefore strongly needed to mine the fast growing number of genome sequences and assess in a rapid and reliable way the pathogenicity of novel bacteria.

**Methodology/Principal Findings:**

We describe a new *in silico* method for the prediction of bacterial pathogenicity, based on the identification in microbial genomes of features that appear to correlate with virulence. The method does not rely on identifying genes known to be involved in pathogenicity (for instance virulence factors), but rather it inherently builds families of proteins that, irrespective of their function, are consistently present in only one of the two kinds of organisms, pathogens or non-pathogens. Whether a new bacterium carries proteins contained in these families determines its prediction as pathogenic or non-pathogenic. The application of the method on a set of known genomes correctly classified the virulence potential of 86% of the organisms tested. An additional validation on an independent test-set assigned correctly 22 out of 24 bacteria.

**Conclusions:**

The proposed approach was demonstrated to go beyond the species bias imposed by evolutionary relatedness, and performs better than predictors based solely on taxonomy or sequence similarity. A set of protein families that differentiate pathogenic and non-pathogenic strains were identified, including families of yet uncharacterized proteins that are suggested to be involved in bacterial pathogenicity.

## Introduction

Bacteria are found in every habitat on Earth, growing in the most different and extreme environmental conditions, including the bodies of live plants and animals. The gut of an adult human contains more than a thousand different microbial species, most of which are innocuous and a few even provide essential functions to their host, from nutrition and development to the regulation of immune responses both in health and disease [1]. However, other bacteria are extremely virulent pathogens with the ability to cause infectious diseases, including cholera (*Vibrio cholerae*), tuberculosis (*Mycobacterium tuberculosis*), tularemia (*Francisella tularensis*), leprosy (*Mycobacterium leprae*) and syphilis (*Treponema pallidum*). When investigating the relationships between bacteria and other living organisms, it is therefore essential to be able to separate pathogenic organisms from non-pathogenic ones. This is complicated by the fact that even the same species might contain both pathogenic and non-pathogenic strains, so that pathogenicity cannot be simply inferred from phylogeny.

A pathogen must have the ability to enter its host, to survive and replicate inside it, and to avoid the normal host cell defenses [2]. It has therefore to be endowed with a set of molecular features comprising a combination of the following classes of factors: *i) adherence factors*, which enable bacteria to attach to a host surface; *ii) invasion genes*, that mediate bacterial entry into eukaryotic cells; *iii) exotoxins*, secreted by the bacteria, which can destroy or affect the function of a host cell; *iv) endotoxins*, that unlike exotoxins are not secreted but are released when the bacterium is lysed; *v)* Several types of *secretion systems* (especially type III and IV), through which toxins can be injected directly from the bacterial cytoplasm into the cytoplasm of its host's cells [3]. Pathogenic *E. coli*, for instance, can cause disease by a large number of different virulence factors that can affect a wide variety of critical host cell processes like protein synthesis, signal transduction, cytoskeletal function, cell division, ion secretion, transcription, apoptosis, and mitochondrial function [4]. A wide variety of virulence factors are found in many combinations in different *E. coli* strains, especially driven by the recombination of these elements through horizontal gene transfer [5], [6]. The important of horizontally acquired features is highlighted by the observation that virulence factors appear to be disproportionately associated with genomic islands [7].

Apart from these features that are directly related to invading and damaging the host, two other classes of genes are important in determining virulence: genes that regulate expression or are required for the activity of “true” virulence factors, and virulence “life-style” genes, acting in the phases of survival inside the host and evasion of the host immune system [8], [9]. Although these genes act only indirectly in determining virulence, they are essential components of the pathogenicity machinery, and their inactivation can attenuate the virulence of a microorganism.

On the other hand, there is also evidence for features that characterize non-pathogenic organisms, the so-called “antivirulence” genes. When an organism becomes pathogenic through a horizontal gene transfer event, some genes may become incompatible with the new lifestyle and they are lost or inactivated through pathoadaptive mutations [10], [11]. These genes are therefore still found in harmless bacteria, but missing or inactive in pathogens.

In traditional studies, the classification of bacteria as pathogens and non-pathogens and the differentiation of pathogens as isolates of high or low virulence have to a large extent been based on the verification of Koch's postulates and, the use of animal models. In more modern studies, we are faced with the fact that most bacterial species are opportunistic pathogens and are hence present also in healthy hosts, making it necessary to verify the pathogenic potential of isolates either in model systems or based on epidemiological studies. Especially when bacterial species or variants are observed for the first time it is very time-consuming and costly to determine their pathogenic potential, and also without guarantee of success since animal models are not always appropriate for describing the analogous biological process in humans. When an unknown bacterium is isolated, it is hence a highly non-trivial and costly procedure to determine its pathogenicity, making the need for in-silico prediction methods apparent. However, the development of such prediction methods cannot only be based on phylogeny, as even the same species might contain both pathogenic and non-pathogenic strains as a consequence of the complex set of features described above characterizing pathogenicity. The need to go beyond phylogeny is emphasized by the extent of horizontal gene transfer (HGT), with portions of genomes exchanged across different species [12].

The increasing evidence of the importance of HGT makes it very challenging to reconstruct a single organismal lineage, with the concept of “species” itself becoming blurred [13], [14]. Traditionally, phylogenetic trees have been constructed on similarity of single genes, in particular the small subunit ribosomal RNA [15], but there is an open controversy about this traditional way of inferring phylogenies [16], [17], also considering that even rRNA is occasionally subject to HGT [18], [19]. New methods rely on whole genome information rather than a single gene, using diverse approaches such as the combination of multiple proteins occurring as orthologs in different organisms [20], [21], conservation profiles [22], protein folds [17], or protein structural domains [23]. What seems to emerge from these studies is the possibility to define a core set of genes that allow reconstructing meaningful phylogenies, but that HGT plays a major role in genome rearrangement and in the eventual ecological diversification of the bacteria, including their pathogenic potential. For example, the comparison of E. coli O157:H7 (a virulent serotype causing haemorrhagic colitis), and non-pathogenic E. coli K-12, reveals that they share a common backbone sequence of 4.1 megabases (Mb), but they also contain respectively 1.34 Mb and 0.53 Mb of introgressed DNA that they do not have in common, characterizing the different lifestyle of the two strains [24].

Several methods have been published aiming at going beyond the simple phylogeny-based approach for the prediction of bacterial pathogenicity. Suen et al. [25] have described a method for predicting the ecological niche of a bacterium, including its potential pathogenicity based on the whole-genome similarity to bacteria with known pathogenicity. This method maps the proteome of each organism on the pre-computed protein families in the Pfam database [26] and uses the Spearman's correlation of these mappings to establish the similarity between bacterial genomes and cluster them into groups. Wu and Moore [27] investigated the correlation relationship between organisms' environmental conditions and gene distribution in certain functional groups, and attempted to predict the environmental conditions from gene content. A number of methods have also been developed for the prediction of virulent proteins, using Support Vector Machine or BLAST alignments to search a database of known virulence factors [28]–[29]. As opposed to these methods, in the approach described here we do not use a set of pre-established families to estimate similarity between organisms, but rather develop a method to select the families which are consistently found only in one of the two types of organisms, pathogens and non-pathogens, and show that this is more powerful than using global similarity. Furthermore, our approach is hypothesis-free and allows us, in contrast to the above methods, to identify new proteins associated with pathogenicity even if they do not have any similarity to any known virulence factor.

The method proposed here seeks to identify features that are distinctive of virulence in microbial genomes of diverse species, and group them in “protein families”. The pathogenicity of query bacteria is predicted based on the presence in their genomes of proteins belonging to these families. The method is developed and benchmarked on a large set of complete bacterial genomes with annotated pathogenicity, and also applied for the prediction of organisms with unknown pathogenicity.

We chose to perform the analysis on the γ-Proteobacteria, a very large and diverse class that comprises many of the most intensively studied bacterial species. It includes human pathogens (*Salmonella enterica*, *Yersinia pestis*), plant pathogens (*Xanthomonas campestris*, *Xylella fastidiosa*), insect endosymbionts (*Buchnera aphidicola*, *Wigglesworthia glossinidia*), as well as a large number of free-living and commensal species. Being so widely studied for its medical and scientific importance, there is a considerable number of complete genomes available for this class, making it well suited for testing our method.

## Results and Discussion

**Figure 1:**
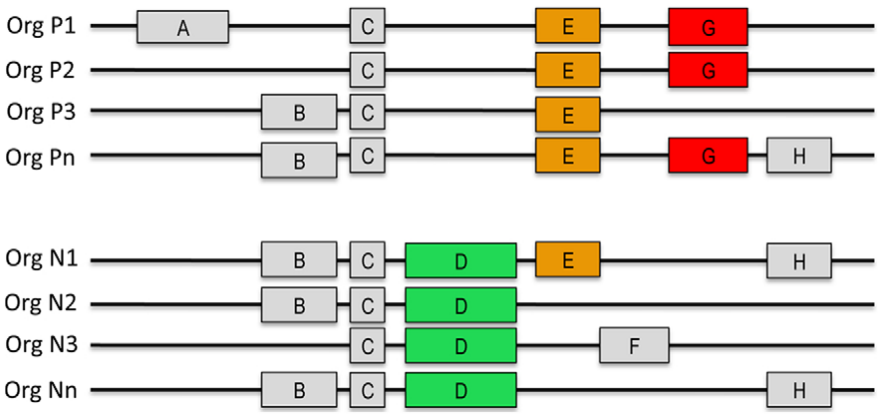
A schematic representation of genomic features shared by pathogenic (P1-Pn) and non-pathogenic (N1-Nn) organisms. Some proteins are only specific of certain strains (A, F), others are shared by different bacteria regardless of their being virulent or not (B, C, H). Proteins that are only (G) or mostly (E) present in pathogenic bacteria (or non-pathogenic bacteria (D)) can be used to discriminate between these two classes, and they might have a role in determining virulence.

The basic idea behind the method was to identify groups of proteins (protein families) that are preferentially present in pathogenic organisms (or non-pathogenic ones) (see **Fig. 1**), and to separate virulent from commensal bacteria, based on the presence or absence of these features. An important distinction to be made here is that we restricted the analysis to γ-Proteobacteria that are potential human pathogens. Thus we considered as pathogenic only organisms reported as able to infect humans, and everything else as non-pathogenic. Families are constructed from protein sequence similarity on a set of γ-Proteobacteria genomes, where any pair of proteins that show a significant sequence alignment E-value are grouped into the same family. Next, significant families are identified based on their size and the proportion of pathogens/non-pathogens they contain (see Materials and Methods). Whether a query organism has proteins contained in these families determines its prediction as either pathogenic or non-pathogenic.

### Predictions on the complete set

The protein families method was optimized to achieve the maximal Matthews correlation coefficient (MCC) in cross-validation, obtaining MCC = 0.748 with 87% of the organisms correctly classified. A value of MCC = 1 indicates a perfect prediction, and a value of MCC = 0 a random prediction. The method was also tested on an independent evaluation set (one fifth of the dataset that was left out in the training phase), and assigned here correctly 16 pathogens out of 17 and 10 non-pathogenic bacteria out of 14, with MCC = 0.682 (84% correctly classified).

### Distribution of predictions across the taxonomy

**Figure 2:**
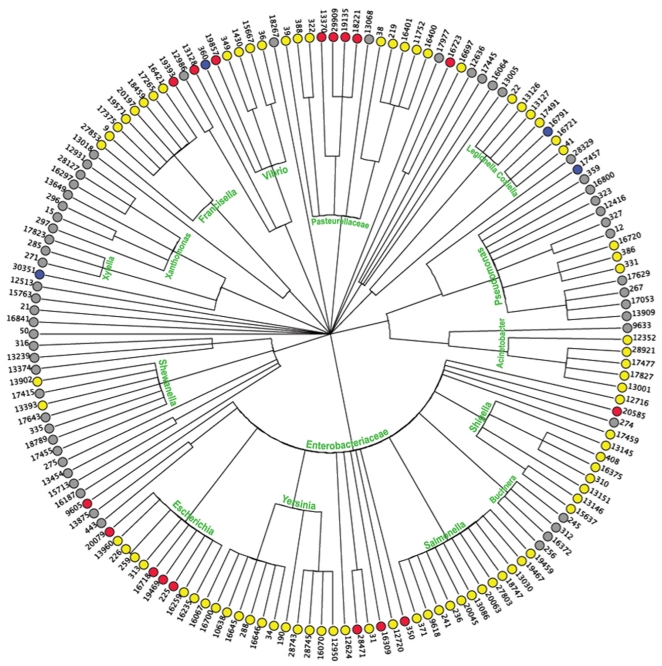
Distribution of the predictions across the taxonomy. The center of the tree corresponds to the class level (γ-Proteobacteria), and the outer levels are in succession: order, family, genus, species. The bacterial lineages were downloaded from NCBI Taxonomy (http://www.ncbi.nlm.nih.gov/Taxonomy/) and the names of the most important clades are reported in green on the figure. The outermost circle displays the single strains in the dataset, labeled with their PID identifier, and colored according to the prediction: Yellow - True Positives, Gray - True Negatives, Red - False Positives, Blue - False Negatives. The figure was produced with the phylogenetic tree viewer Dendroscope [46] with manual annotation of the clade names.

**Fig. 2** shows how the predictions are distributed relatively to taxonomy. Some genera seem much easier to predict, namely those that in this dataset are only composed of pathogens (like *Yersinia* or *Legionella*) or non-pathogens (like *Buchnera*, *Xanthomonas*). Others show a more variegated picture and are also the most difficult to assign. Whereas for e.g. *Shewanella* virulent and avirulent strains are correctly separated, the results for *Escherichia* are very poor: the method predicts all 10 strains as pathogenic although 4 of them are not. This is most likely due to the high degree of sequence similarity between the *E. coli* genomes in the dataset, making the large number of features they have in common overcome the few ones that discriminate the pathogenic strains from the others, even though the method tries to only detect the latter.

By retraining the method only on the members of the *Enterobacteriaceae* (which comprises *E. coli*), more subtle differences between the proteomes can be detected. Whereas all the other predictions for *Enterobacteriaceae* remained unchanged, 2 of the wrongly assigned *E. coli* were corrected using the reduced dataset. The correlation coefficient on this set was MCC = 0.676 in cross-validation, and MCC = 0.770 on the test-set. Restricting the analysis to a branch of the complete dataset is possible only when a sufficient number of organisms is present in the subset to train the method on. The minimum size of a subset was estimated by running the predictor on reduced sets of *Enterobacteriaceae* from the original dataset of 58 organisms. The performance in cross-validation drops to MCC = 0.455 with 40 organisms, 0.275 with 30 organisms, and becomes close to random with 20 organisms (MCC = 0.135).

### Investigation of the species bias

Within the same class of bacteria, one can find a wide range of organisms causing diverse diseases in different hosts, as well as many non-virulent ones. However, it is also true that they do not distribute evenly across the taxonomy, and some clades are highly homogeneous and composed mainly of pathogens or mainly of avirulent strains (see **Supplementary Fig. S1** online). These subgroups are the easiest for the predictor to assign correctly, as they have many proteins in common and are easily clustered together. The task is more complex on clades that comprise both pathogenic and non-pathogenic organisms, because the predictor should be able to only individuate the features that characterize virulence, and separate the organisms upon the presence or absence of these features.

The extent of the species bias can be estimated by comparing our method to one solely based on taxonomy. Such a model simply determines whether the closest relatives (in terms of taxonomy classes, see *Materials and Methods*) of the query organism in the dataset are pathogenic or not, and classifies the query accordingly. The performance of this method on 10,000 bootstrapped datasets was MCC = 0.571, with standard deviation SD = 0.047. On the same datasets the protein families method yielded MCC = 0.722 (SD = 0.038), thus outperforming the taxonomy-based predictor with p = 0.002. Our method was also compared to one based on global sequence similarity, using the BLAST alignment bitscores as illustrated in *Materials and Methods*. In the same way the performance of the protein family-based method resulted significantly better than the alignment-based predictor with p = 0.014 (MCC = 0.620, SD = 0.045).

Global relatedness and position in the taxonomy are clearly important factors in the distribution of pathogens. On the other hand we have here shown that pathogenicity is often characterized by a relatively small number of genes, so that two organisms can have similar genomes at a global sequence level and only differ for these few key features that discriminate virulent and avirulent bacteria. In the case of *E. coli*, for instance, the acquisition of a single pathogenicity island can be enough to transform a symbiotic strain into a virulent one [3]. The classification of *E. coli* strains into pathogenic and non-pathogenic suggests that the predictions are strongly dependent on the taxonomic level used in the training (in this case, *class* vs. *family*), and that finding the correct level is of major importance to detect subtle differences between very similar genomes, and separate the pathogenic from the non-pathogenic ones.

### Validation on other independent data sets

The predictive power of the method was evaluated on a set of 24 genomes from diverse branches of the γ-Proteobacteria class, released after the main dataset for training was assembled. The data set contains 14 organisms annotated as pathogenic and 10 as non-pathogenic. The predictor assigned correctly 22 out of 24 organisms (91.7%), with a MCC of 0.837. One of the wrongly predicted organisms is *Haemophilus parasuis* SH0165 (NCBI project ID (PID) 31099), a pathogen of swine that causes the severe systemic disease known as Glasser's disease [30]. The other is *Salmonella enterica* Serovar Gallinarum str. 287/91 (PID 30689), a strain that causes typhoid in poultry, but is not pathogenic for humans. *S.* Gallinarum has been suggested to be a recently evolved descendent of *S. Enteritidis*, which is a host-promiscuous serovar of *Salmonella enterica* that can also infect humans. Although the virulence of *S.* Gallinarum is restricted to chicken, this strain retains a good number of genomic regions from its human-infecting ancestor, including many of the *Salmonella* pathogenicity islands [31]. Probably the presence of these features misleads the predictor into considering the organism as a pathogen in human.

Another evaluation set was composed of organisms that were initially excluded from the analysis, as NCBI Genome Project does not annotate them as either pathogenic or non-pathogenic. The set contains 27 organisms, with a prevalence of the genera *Shewanella* (10 strains) and *Escherichia* (6 *E. coli* strains), and a variety of other species belonging to the γ-Proteobacteria class. Firstly, using the complete dataset for training, all the *E. coli* in this dataset are predicted as pathogenic. As observed previously, subtler differences can be detected by restricting the dataset to the family level, if enough genomes are available for a particular family. This is the case of the *Enterobacteriaceae* group, which contains 58 different organisms in the main dataset. Thus, the analysis was repeated only using the *Enterobacteriaceae* dataset for the training, and the same parameters that were found to be optimal in cross-validation. With this approach, the 7 *Enterobacteriaceae* were now separated into pathogenic and non-pathogenic, with 5 falling into the first category and 2 into the latter. The two bacteria predicted as non-pathogenic are two serovars of *E. coli* K-12 (W3110, PID 16351 and ATCC 8739, PID 18083), a strain widely used in laboratory experiments for its safety and easiness to grow. It is normally avirulent, as are also two other strains of K-12 in the main dataset (MG1655, PID 225 and DH10B, PID 20079).

### Analysis of the protein families

A very interesting by-product of the method is the set of protein families that is built for the prediction. These families are composed of proteins that discriminate pathogenic from non-pathogenic organisms, and might point out interesting genomic features that are related to virulence.

If a particular gene is consistently present in pathogens but absent in non-pathogenic strains (or conversely, consistently present in non-pathogenic bacteria but absent in pathogens), then there is a high probability that this particular gene is involved in processes that are typical of the lifestyle of a pathogen (or non-pathogen). The strength of this approach is that it potentially does not only identify toxins or other strict virulence factors, but also genes that are connected to their regulation in some way, and a thorough analysis of the protein families might potentially reveal some unknown relationships of this sort.

On the current dataset, 381 families met the criteria of “pathogenicity family”. The most common known functions of members of these families are “exported proteins” (32 families) and “membrane proteins” (30 families), but also other classical virulence factors emerge as overrepresented in pathogenicity families such as “secretion systems” (16 families) and “fimbrial” and “flagellar” proteins (respectively 11 and 6 families). On a random sample of 381 protein families, the same functions were found in the following number of families; 4 exported, 12 membrane proteins, 1 secretion system, 4 fimbrial, 3 flagellar. The families were built with no prior knowledge about the known function of their members, thus recovering a strong association of well-established virulence factors with blindly-built pathogenicity families supports the validity of the approach.

In **Table 1** are listed the functions of the proteins that are found in the 10 top-scoring families, ranked according to Z-scores as described in *Materials and Methods*. Some of these 10 families contain proteins that are clearly involved in pathogenicity. The members of families rank 2 and 7 are fundamental for bacterial adherence, a crucial step in the colonization of a new host. Pili and fimbriae are in this class of proteins, and are hair-like appendices that provide bacteria with an efficient mechanism to attach to host surfaces. Other molecular attributes that can easily be linked to virulence are type III secretion system components (family rank 10), used by bacteria to secrete directly from the bacterial cell to the host, and heat shock proteins (family rank 8), which can be important for the survival of a bacterium right after it has entered its host.

**Table 1:** 10 top scoring pathogenicity families, and function of their members.

Rank	Z-score	P	N	Function of proteins in the family
1	8.29	42	4	Mutarotases, YjhT proteins
2	8.25	33	1	Fimbrial proteins, putative adhesins
3	8.12	38	3	Proteins of unknown function
4	8.02	40	4	Cytochrome b_562_
5	7.89	39	4	Proteins of unknown function
6	7.86	36	3	Methyltransferases
7	7.82	30	1	Fimbrial proteins, pilin proteins
8	7.56	25	0	Heat shock proteins, DNA-repair
9	7.46	36	4	5-carboxymethyl-2-hydroxymuconate isomerase
10	7.06	25	1	Type III secretion proteins, path. island proteins

Family rank 1 contains YjhT proteins, a family of proteins that are present in many sialic acid utilizing pathogens. The presence of sialic acid onto bacterial cell surfaces is thought to allow pathogens to disguise themselves as host cells and elude immune response [32]. Family rank 4 groups cytochrome b_562_ proteins from various different organisms. Cytochromes in bacteria are suggested to provide some sort of protection against chemical attacks from reactive species, such as reactive oxygen and nitrogen species, and allow survival and growth in oxygen-limited conditions [33]. Methylation of DNA, operated by methyltransferases (family rank 6), is an important process in bacterial cells that affects the regulation of transcription, chromosome replication, DNA segregation, mismatch repair and transposition. Further, it is emerging from various studies that DNA methylation has a role in regulating the expression of various bacterial genes related to virulence in several pathogens [34], and methyltransferase genes have been found on pathogenicity islands [35]. 5-carboxymethyl-2-hydroxy-muconate isomerase (family rank 9) is an enzyme that transposes C = C bonds and catalyzes metabolic reactions [36]. There is no apparent reason why this enzyme should be involved in pathogenicity.

Finally, two families (rank 3, and 5) are only composed of proteins with unknown function. They are potentially even more interesting than well-characterized virulence factors, such as toxins or fimbriae, as they might turn out to be molecular components of bacterial virulence apparatuses that are still completely unknown. A large number of genes, in fact, still have unknown function even though they are highly conserved among bacterial genomes [37]. Family rank 3 is entirely composed of proteins with unknown function from 41 different organisms, 38 of which (92.7%) are pathogens, and scrolling down the list of families with lower but still significant rankings many others are found only composed of uncharacterized proteins.

**Figure 3:**
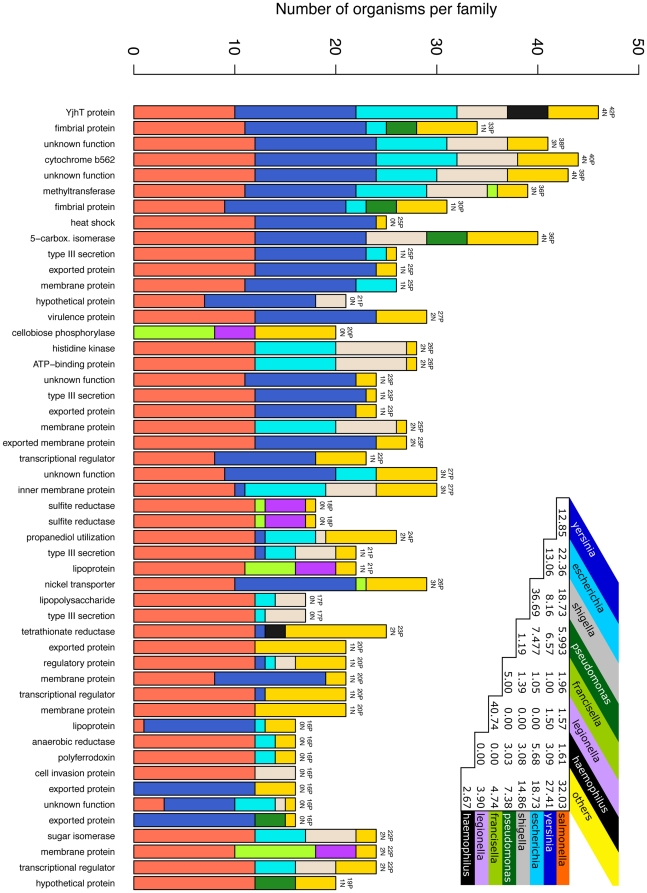
Genera composition of the 50 top scoring pathogenicity families. Vertical bars in the plot represent single families, where the height of each bar is the number of organisms per family, and the numbers on top of the bars indicate the proportion of pathogens vs. non-pathogens. Each color represents a bacterial genus, according to the color scheme of the table in the top-right corner. The table summarizes the frequency of co-occurrence of any pair of genera A and B, where the frequency is calculated as the number of families containing A and B, divided by the number of families containing both A and B (the values are given as percentages).

In terms of species composition of the protein families, we observed that 32% of the families were constituted of proteins from only one bacterial genus. However, all the largest and most significant pathogenicity families contained proteins from two and often more genera (Fig. 3), where also some interesting trends of co-occurrence of different organisms were observed. In particular, *Escherichia* and *Shigella* strains were found together in 37% of the families that contained either of these two genera (see table in Fig. 3), as can be expected from two such closely related organisms. *Shigella* strains are actually often just considered as belonging to the *Escherichia coli* species [38]. More unexpected was the high frequency of co-occurrence of *Francisella* and *Legionella*, as phylogenetically the *Francisellaceae* are a quite separated family with no close relatives. However, among human pathogens *Legionella* species (together with *Coxiella*) are the most closely related to *Francisella*, and they share similar lifestyles [39], [40]. *Salmonella* and *Yersinia* are present in many pathogenicity families, often together, and some of the most significant families contain up to all 12 *Salmonella* strains and all 12 *Yersinia* strains in the dataset.

### A case story: protein family 6758

**Figure 4:**
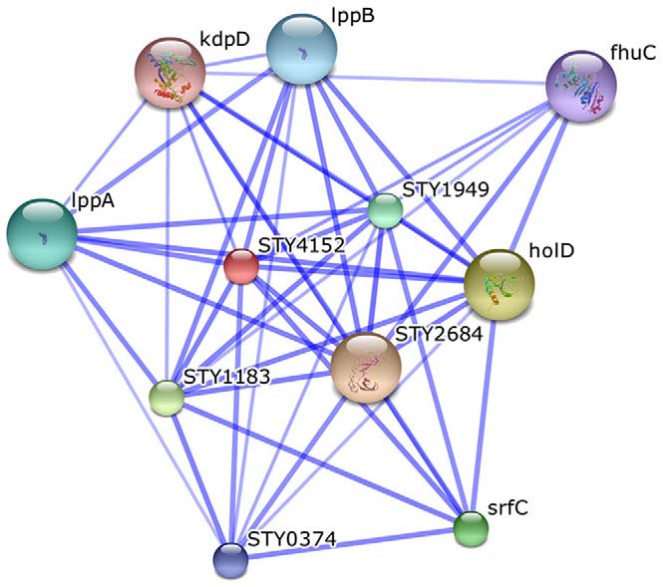
Interaction network for protein STY4152 from Salmonella enterica CT18. STY4152 is annotated as “hypothetical protein” and is assigned to the pathogenicity family 6758 by the protein families method. The predicted functional partners are: STY2684 (putative lipoprotein); holD (DNA polymerase III subunit psi); STY1183 (hypothetical protein); srfC (putative virulence effector protein); STY1949 (putative lipoprotein); lppA (major outer membrane protein); lppB (major outer membrane protein); STY0374 (possible transmembrane regulator); fhuC (ferrichrome transport ATP-binding protein FhuC); kdpD (sensor protein KdpD). The thickness of the connection lines represents the degree of confidence of the interaction. Image from the STRING database [43].

Family 6758 is detected by the method as significant for pathogenicity (rank 18), and contains yet uncharacterized proteins. They come from 24 different γ-Proteobacteria, 23 of them pathogenic (*Salmonella* spp. and *Yersinia* spp.) and one non-pathogenic (*Pectobacterium atroseptisum*, PID:350), with sequence length between 179 and 195 amino acids, all annotated as hypothetical proteins. A multiple sequence alignment shows that the main part of the sequence is almost identical for all proteins, and differences are observed only in the first 30–35 AAs. Specifically, there seem to be two different kinds of N-terminal sequences, one for *Yersinia* strains and one for *Salmonella* strains, both predicted to be signal peptides by SignalP 3.0 [41]. Their subcellular localization is predicted by PSORT [42] to be the outer membrane for the *Yersinia* version, and either outer membrane or periplasmic space for *Salmonella*. A search for associations in the STRING database [43] indicates that proteins in family 6758 co-occur, among others, with virulence factors and enterotoxins (in **Fig. 4** is shown the interaction network for one representative protein in this family).

### Conclusions

There is a strong need for better data-mining algorithms in the fast growing body of genomic information. This work focuses on such a need, presenting a new, reliable method for the prediction of bacterial pathogenicity, based on the bioinformatics-based identification of features in microbial genomes that appear to correlate to virulence. The method was applied here to a large dataset of γ-Proteobacteria complete genomes, and it was demonstrated that this approach goes beyond the species bias imposed by evolutionary relatedness, and perform better than predictors that only rely on taxonomy and global sequence similarity. Furthermore, we observed that the quality of the predictions improves as the number of genomes used for training the method increases, promising enhanced performance as more complete genome sequences become available.

The novelty of this approach lies in the fact that no prior knowledge about protein function is used to identify features that correlate with pathogenicity (like for instance virulence factors), but rather inherently builds families of proteins that are consistently found in pathogenic organisms, regardless of their known function, and uses those for the predictions. These families associated with pathogenicity include groups of proteins that are functionally uncharacterized, but hence underlined by the method as potential players in defining bacterial virulence as well as targets for antimicrobial drugs and vaccines.

## Methods

### Pathogenic and commensal γ-Proteobacteria

The analysis was performed on the γ-Proteobacteria class, a large and diverse group that comprises lots of medically important organisms. Being so widely studied, there are many genome projects already completed or under way for this class, including human pathogens (*Salmonella*, *Yersinia*), plant pathogens (*Xanthomonas*, *Xylella*), insect endosymbionts (*Buchnera*, *Wigglesworthia*) and a vast number of commensal species. Out of the 182 organisms with a complete sequence (NCBI Genome Project, http://www.ncbi.nlm.nih.gov/genomes/lproks.cgi, accessed on 30 October 2008), 83 were annotated as able to infect human, 72 as non-pathogenic for human, and 27 lacked annotation so they had to be excluded from the analysis.

We considered as human pathogens all those organisms that were reported having as host either human, mammal or animal, the latter two classes also comprising the human species. The “non-pathogenic” included all the bacteria marked as non virulent, as well as pathogens of plant, insect, fish and any other non human species. Plant pathogens, for instance, have likely a very different invasion strategy than pathogens of human, and therefore also a very different set of virulence genes. More controversial is the case of hosts like porcine, since their pathogens can have some features in common with human ones to some extent. With the proposed classification criterion they are just considered as non-pathogenic, but the molecular features they possibly share with human pathogens might make the prediction more difficult.

### Datasets assembly

The full amino acid sequences of complete genome projects for γ-Proteobacteria available to date (30 October 2008) were obtained from GenBank, and the coding regions of the genomes, comprising also eventual plasmids, were extracted. The NCBI genome project [44] assigns to each organism that has been sequenced a numerical identifier (project ID), and reports whether the organism is pathogenic (**P**) or non-pathogenic (**N**). The information about diseases and hosts of γ-Proteobacteria in the dataset was extracted using html-parsing from the NCBI website. Those with uncertain or unknown pathogenicity were removed from the dataset and not considered in the initial analysis.

The pool of bacterial genomes was split into 5 subsets, selecting the organisms randomly within each of the two classes **P** and **N**, but making sure that each subset had the same proportion of pathogenic and non-pathogenic bacteria. One of these 5 subsets was set aside as an independent test set, while the other 4 parts were used for cross-validation when developing the algorithms.

A database, comprising all the coding regions of the organisms under investigation, was then constructed and preprocessed so that it could be searched by BLAST-methods. After this operation, an all-against-all BLAST search [45] was performed by applying blastpgp for all the proteins in the database against the database itself. A cutoff of 10^−20^ on the E-value, and a maximum number of 500 hits per input protein, has been applied to limit the size of the results file. A larger number of hits would introduce mostly redundant data and penalize greatly the computation time. Even reducing this number from 500 to 100 leads to a loss of only 0.16% of the protein families, and does not affect the predictions.

### Construction of the protein families

Clusters of similar proteins, that we will call “protein families”, are built from the BLAST results. Any two protein sequences which align with a significant degree of similarity (E-value<10^−20^) will fall in the same protein family. The protein space can be visualized as a graph-like structure, where nodes represent proteins and a significant alignment is saved as an edge between two nodes. Once the graph structure is built, and all proteins and relationships between them are stored, a graph traversal algorithm explores it and identifies the connected subsets of the graph. For any pair of proteins F and G, if there exists a path in the graph connecting them, they belong to the same protein family. This is equivalent to saying that connected subsets of the graph represent protein families.

The function annotation of each protein is extracted from the “\product” field of the protein's CDS in the GenBank file, removing from the annotation symbols and common words such as “probable”, “putative”, “conserved” and others, to obtain a standard dictionary of meaningful descriptions. The consensus of the functions thus derived for each protein determines the function associated to the families. For the top ranked families discussed in the results manual curation was also performed to ensure optimal annotation of the function.

### Scoring of significant families and prediction of a query organism

Significant protein families are identified following two criteria: first, the number of organisms (*ORG*) which have proteins in the family has to be bigger than a certain value (*NP*) for the cluster to carry enough information; second, the ratio of the number of pathogens having proteins in the family on the total number of organisms in the family (*P_ratio_*) has to be higher than a given threshold (*HG*) for the cluster to be considered as a “pathogenicity family”, or on the other hand smaller than a low number (*LW*) to be mainly composed of proteins from non pathogenic organisms. These quantities are taken into account to calculate a score for each family according to a double-step function:
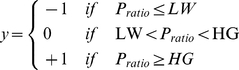
(1)if *ORG *≥ *NP*, and *y* = 0 otherwise.

The proteome of a given query organism is scanned to detect which of its proteins fall into significant families, and their scores are summed up. If the total is bigger than zero then the prediction is **P** (pathogenic), otherwise the prediction is **N** (non-pathogenic).

### Models based on taxonomy and global similarity

A simple classifier based solely on taxonomy was designed to estimate the extent of the species bias. It assigns a bacterium to the **P** or **N** class according to the majority of its nearest relatives, i.e. if the closely related organisms are mostly pathogenic or not. Starting from the species level, if the dataset contains other bacteria of the same species as the query, it computes the proportion of pathogens/non-pathogens in this part of the tree, and assigns the query according to the majority. If there is a tie, or there are no organisms of the same species, the algorithm moves up one step to the genus level. Again, the proportion of pathogens in the clade determines the classification of the query, going up one level at a time until a prediction can be made, with the class level as the extreme case (the whole dataset).

Another null model, based on global sequence similarity, exploits the BLAST alignment bitscores. The bitscore is a measure of the quality of the alignment, also accounting for the length of the sequence overlap, and any gaps that have to be introduced to align the sequences. In this method, for any given proteome-proteome pair in the dataset, the bitscores of all protein-protein BLAST matches are summed up, and an average is calculated on the length of the query proteome. The query is classified as P or N according to the organism with the highest average bitscore.

The performance of both the above models was compared to the protein families method using a bootstrapping technique. Multiple datasets were built by dividing in 10,000 alternative ways the original dataset into 4 subsets, and all three methods were run in cross-validation on these new 10,000 datasets. For each, a performance value in terms of MCC was calculated, and we determined the fraction of datasets where the null models have higher MCC than the protein families method. This is considered as a p-value for the protein families method to have significantly better performance.

### Cross-validation to prevent overfitting

The optimal parameters were chosen using a 4-fold cross-validation, i.e. by assessing the performance of the predictor using a portion of dataset (one fourth) that was not included in the training phase. This is repeated for 4 times utilizing each time a different fourth of dataset for testing. The test-sets are then pooled to form a complete set of predictions, and the optimal parameters are chosen on this set. The peak in performance is obtained with *HG* = 0.85, *LW* = 0.04, *NP* = 6.

The optimized version of the method was eventually tested on a completely independent evaluation set, that was left aside in the analysis up to this point, and also on other additional datasets. They were not included in the training, therefore they provide an unbiased evaluation of the method's performance.

### Z-scores for the ranking of protein families

The significance of a protein family depends on two factors: its size *ORG* and the ratio of pathogens on the total number of organisms it contains (*P_ratio_*). A statistical measure, the Z-score (*Z*), is designed to take both these indices into account. The population of families used to calculate *Z* is reduced to only those with *ORG *≥* NP*. On this set it can be estimated which families have a *P_ratio_* that deviates in a significant manner from the mean ratio of the other families. The value of *Z* is a unitless measure representing by how many standard deviations the mean *x* of the sample (i.e. a protein family) is different from the mean *μ* of the population, and is calculated as:
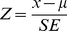
(2)where the standard error of the mean *SE* is:
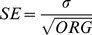
(3)


The mean μ represents the average of *P_ratio_* of all families in the population, and *σ* is the standard deviation of *P_ratio_* across these clusters. Together, *μ* and *σ* permit to draw a distribution of the ratios, so that one can infer if the *P_ratio_* of a given family deviates significantly from the average.

## Supporting Information

Figure S1 Phylogenetic tree of the 155 organisms in the main dataset. The root corresponds to the class level (γ-Proteobacteria), and moving to the right towards the single strains the levels are order, family, genus, species, subspecies. Pathogenic and non-pathogenic strains are depicted in different colors (Red: pathogenic, Blue: non-pathogenic) and show how virulent organisms distribute across the taxonomy.
(1.19 MB PDF)
