# Supplementary figures and images for: In Silico Prediction of Human Pathogenicity in the γ-Proteobacteria
Source: PLoS One. 2010 Oct 27;5(10):e13680. doi: 10.1371/journal.pone.0013680 (PMC2965111; doi:10.1371/journal.pone.0013680)

Gamma-Proteobacteria

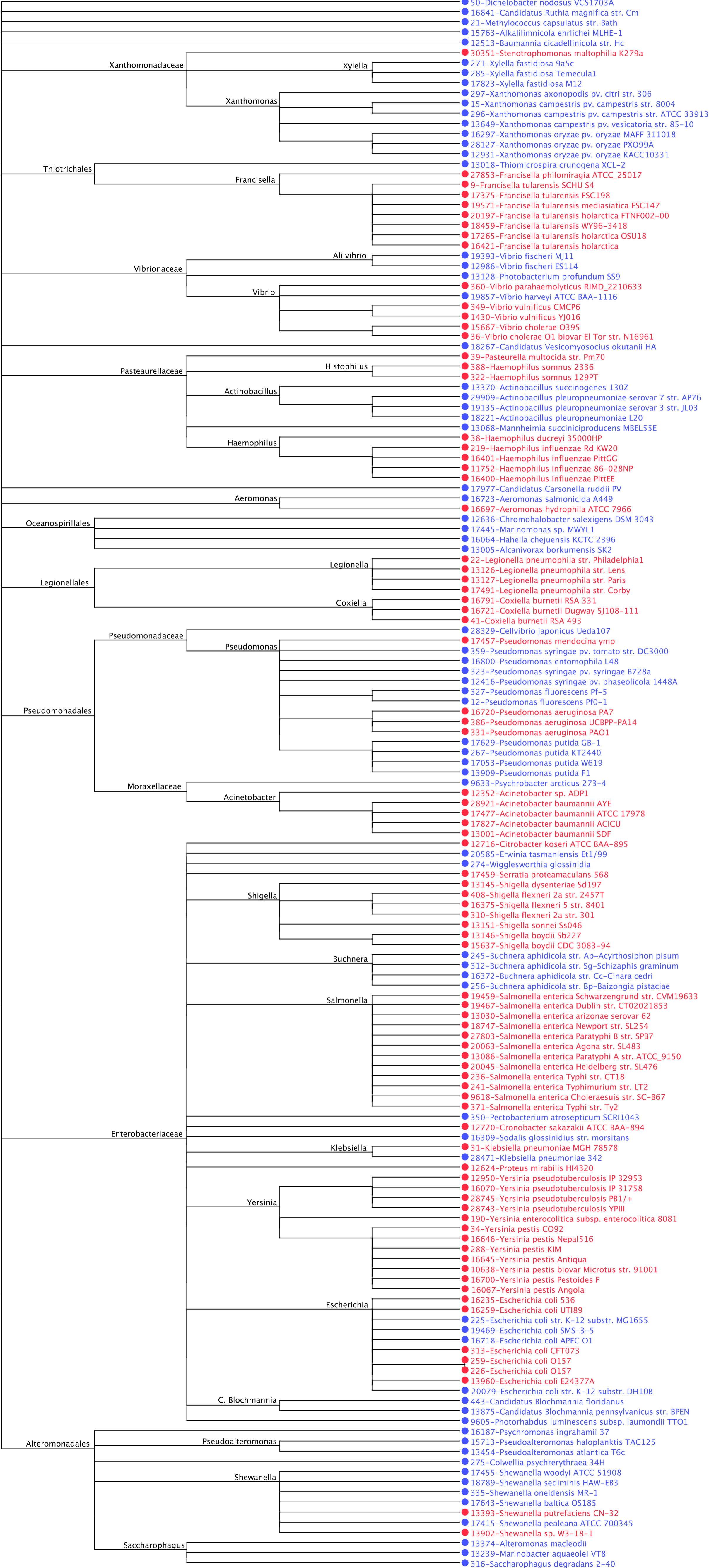

Supplement: Figure S1 — Phylogenetic tree of the 155 organisms in the main dataset. The root corresponds to the class level (γ-Proteobacteria), and moving to the right towards the single strains the levels are order, family, genus, species, subspecies. Pathogenic and non-pathogenic strains are depicted in different colors (Red: pathogenic, Blue: non-pathogenic) and show how virulent organisms distribute across the taxonomy. (1.19 MB PDF) [file pone.0013680.s001.pdf]
